# Immunofluorescence Studies on the Expression of the SARS-CoV-2 Receptors in Human Term Placenta

**DOI:** 10.1159/000521436

**Published:** 2021-12-16

**Authors:** Jürgen Becker, Danny Qiu, Walter Baron, Jörg Wilting

**Affiliations:** ^a^Department of Anatomy and Cell Biology, University Medical School Göttingen, Göttingen, Germany; ^b^Department of Gynecology, Agaplesion Hospital Neu Bethlehem, Göttingen, Germany

**Keywords:** Placenta, Syncytiotrophoblast, Stromal cells, Hofbauer cells, Endothelial cell, Angiotensin-converting enzyme 2, TMPRSS2, Neuropilin 1, TEK tyrosine kinase, CCBE1, Vimentin

## Abstract

Until September 2021, the Severe Acute Respiratory Syndrome Coronavirus-2 (SARS-CoV-2; COVID-19) pandemic caused over 217 million infections and over 4.5 million deaths. In pregnant women, the risk factors for the need of intensive care treatment are generally the same as in the overall population. Of note, COVID-19-positive women deliver earlier than COVID-19-negative women, and the risk for severe neonatal and perinatal morbidity and mortality is significantly higher. The probability and pathways of vertical transmission of the virus from the pregnant woman to the fetus are highly controversial. Recent data have shown that 54 (13%) of 416 neonates born to COVID-19-positive women were infected. Here, we investigated term placentas collected before the SARS-CoV-2 pandemic and studied the main COVID-19 receptors angiotensin-converting enzyme 2 (ACE2), transmembrane protease serine subtype 2 (TMPRSS2), as well as neuropilin 1 (NRP1). We performed real-time PCR and immunofluorescence on cryosections in combination with markers for syncytiotrophoblast, endothelial cells, macrophages and stromal cells. The PCR studies showed expression of both the truncated delta form of ACE2, which does not bind the COVID-19 spike protein, and the long form. The ACE2 antibody used does not distinguish between the two forms. We did not observe expression of the canonical SARS-CoV-2 entry machinery on syncytio- and cytotrophoblast. ACE2 and TMPRSS2 are co-expressed in a subpopulation of stromal cells, which in part are CD68-positive macrophages. NRP1 is localized to endothelial cells. In sum, the term placenta is not an organ that directly favors vertical transmission of COVID-19; however, microtraumas and placentitis may weaken its barrier function.

## Introduction

As of August 31, 2021, the World Health Organization has confirmed over 217 million infections and over 4.5 million deaths caused by Severe Acute Respiratory Syndrome Coronavirus-2 (SARS-CoV-2; COVID-19) [https://www.arcgis.com/apps/dashboards/bda7594740fd40299423467b48e9ecf6). Similar to the overall population, in pregnant women, pre-existing comorbidities, high maternal age, and high body mass index are among the risk factors for the need of intensive care treatment [Allotey et al., 2020]. Thereby, vertical transmission to the embryo or fetus is a matter of great general interest. Villar et al. studied vertical transmission of COVID-19 and compared 706 pregnant women with COVID-19 to 1,424 pregnant women without COVID-19. Of note, COVID-19^+^ women delivered earlier than COVID-19^-^ women, and the ones with symptoms 1.5 days earlier than the ones without symptoms, after approx. 30 weeks' gestation [Villar et al., 2021]. The risk for severe neonatal and perinatal morbidity and mortality was significantly higher in the group of COVID-19^+^ women. 416 neonates born to COVID-19^+^ women were tested for the virus, and of these, 54 (13%) were COVID-19^+^. Thereby, cesarean delivery may have increased the risk for neonates to test positively (RR, 2.15; 95% CI, 1.18–3.91), and Villar et al. emphasize that contamination during cesarian resection has to be taken into consideration. In a previous review on 324 pregnant women with COVID-19, only 3 of 155 neonates tested were COVID-19^+^ [Juan et al., 2020]. In a living systematic review (last update Nov. 29, 2020) it is noted that “existing evidence has not identified major risks of complications in babies born to mothers with COVID-19” [Yap et al., 2020]. More recent studies confirmed increased risks of preterm delivery and maternal mortality caused by COVID-19 infection [Mullins et al., 2021]. Transfer of anti-COVID-19 IgG from mother to fetus is very likely, but IgM antibodies, which are unlikely to pass the placental barrier, have also been detected in some newborns [Zeng et al., 2020; Dong et al., 2020].

Considering how close the connection between mother and fetus is, vertical transmission of COVID-19 appears to be relatively low. There are, however, a number of studies that point to the existence and significance of COVID-19 receptors in placenta. Immunoperoxidase studies showed focal expression of the main COVID-19 receptor angiotensin-converting enzyme-2 (ACE2) on placental syncytiotrophoblast [Hikmet et al., 2020], immediately at the feto-maternal interface. Additionally, with analyses of the GEO scRNASeq database, Lü et al. found expression of ACE2 in 9 out of 9,852 cells derived from one placenta, which were also GATA3-positive and therefore classified as trophoblast cells (not differentiating between cyto- and syncytiotrophoblast) [Lü et al., 2020]. The authors took this as an indication that maternal COVID-19 may directly infect trophoblast cells. Studying single-cell and single-nuclear RNA-sequencing data, Pique-Regi et al. [Pique-Regi et al., 2020] concluded that the number of placenta cells coexpressing ACE2 and the serine protease TMPRSS2, which is widely accepted as the most important activator for cell entry of SARS-CoV-2 [Hoffmann et al., 2020], is negligible, making vertical transmission highly unlikely. In contrast, with paraffin histology, expression of ACE2 protein and SARS-CoV-2 spike RNA in multiple parts of placenta and umbilical cord have been described [Verma et al., 2021]. However, the effects of boiling tissues in a microwave, as in these studies [Verma et al., 2021], are not necessarily predictable.

Here, we investigated term placentas collected in 2015 before SARS-CoV-2 pandemic and studied ACE2 and TMPRSS2 by real-time PCR (qPCR) and Western blot. We then performed immunofluorescence (IF) on cryosections to study the cellular localization of these molecules in addition to neuropilin-1 (NRP1), which can assist SARS-CoV-2 infection [Li and Buck, 2021], in combination with markers for syncytiotrophoblast, endothelial cells, macrophages, and stromal cells. We did not observe evidence for the expression of SARS-CoV-2 entry molecules on syncytio- and cytotrophoblast. ACE2 and TMPRSS2 are found in stromal cells, which in part are CD68-positive macrophages. Our qPCR analyses showed expression of the long and the truncated (delta) isoform of ACE2, which does not bind to the viral spike protein [Onabajo et al., 2020]. Under physiological conditions, the term placenta appears to provide a barrier against SARS-CoV-2 infection.

## Materials and Methods

### Tissues and Cells

Six term placentas were collected after vaginal births of healthy children with the informed, written consent of the mothers. Specimens were collected in 2015 clearly before the start of the COVID-19 pandemic, and stored at −80°C. Specimens were rinsed in phosphate-buffered saline (PBS) and freshly frozen for Western blot analysis and real-time PCR, or fixed for histology and immunohistology in 4% paraformaldehyde overnight. The studies were approved by the ethics committee of the University Medical Hospital Göttingen, UMG (application no. 18/1/18), and methods were performed in accordance with the relevant guidelines and regulations. For comparative Western blotting and qPCR, human lymphatic endothelial cells were purchased from PromoCell (Heidelberg, Germany), cultured in LEC medium and checked for purity, as described recently [Blesinger et al., 2018]. Various human tumor cell lines were cultured under standard conditions as described before [Becker et al., 2010].

### IF Studies

Specimens were rinsed in PBS, transferred into 10 and 30% sucrose in PBS, and embedded in tissue freeze medium (Tissue Tek, Sakura Finetek Zoeterwoude, NL). Sections of 12 µm were incubated with the primary and secondary antibodies listed in Table 1. Sections were counter-stained with DAPI, and mounted under coverslips with Fluoromount-G (Southern Biotechnology, Birmingham, AL, USA). Photos were taken with AxioImagerZ1 (Zeiss, Göttingen, Germany).

### Real-Time RT-PCR

Real-time RT-PCR (qPCR) was performed as described [Becker et al., 2021]. Primers are listed in Table 2.

## Results

**Fig. 1:**
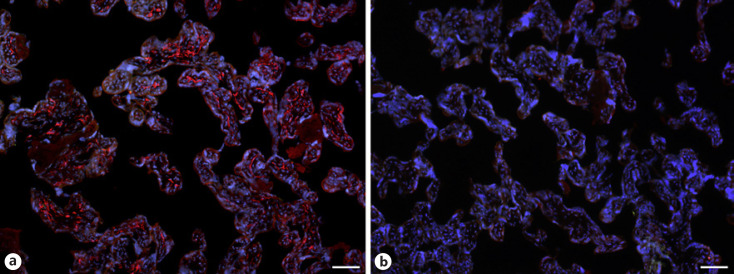
**a** Immunofluorescence picture showing ACE2 (red) in term placenta. **b** Control without primary antibody. ACE2 is found in stromal cells. Nuclei are stained blue with DAPI. Bar = 80 µm.

**Fig. 2:**
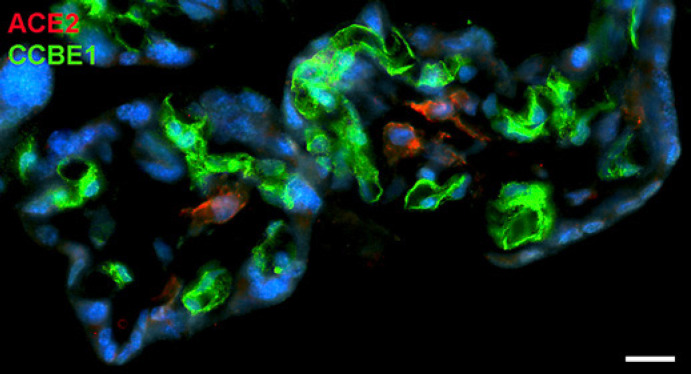
Immunofluorescence picture showing ACE2 (red) and CCBE1 (green) in term placenta. CCBE1 demarcates the capillaries and ACE2 stromal cells. Nuclei are stained blue with DAPI. Bar = 20 µm.

**Fig. 3:**
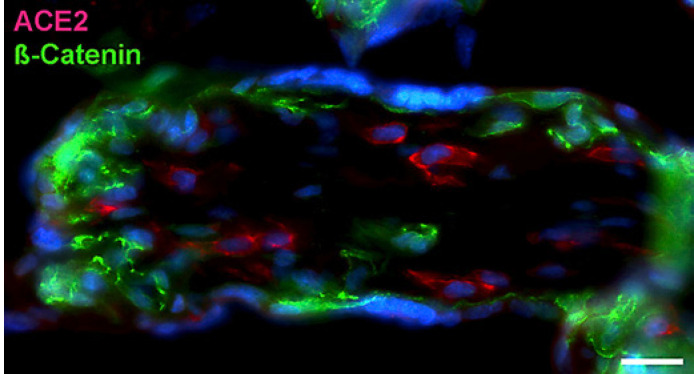
Immunofluorescence picture showing ACE2 (red) and β-catenin (green) in term placenta. β-Catenin demarcates mainly the basal aspect of the syncytiotrophoblast. ACE2 is found in stromal cells. Nuclei are stained blue with DAPI. Bar = 25 µm.

**Fig. 4:**
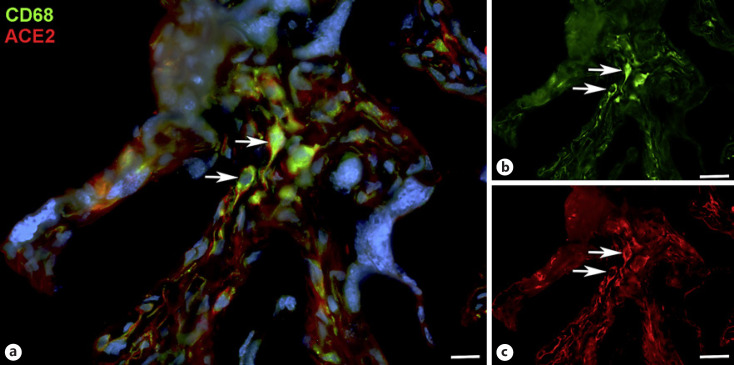
Immunofluorescence picture showing ACE2 (red) and macrophage marker CD68 (green) in term placenta. **a** Merged picture; **b** and **c** show each channel separately. A subpopulation of ACE2+ cells can be identified as macrophages (arrows). Nuclei are stained blue with DAPI. Bar = 20 µm in A, and 40 µm in B, C.

With qPCR, we found clear signals for both ACE2 and TMPRSS2 in term placenta. ACE2 exists in various isoforms ranging from 805 aa (92.5 kDa; canonical isoform) to 459 aa (52.7 kDa; short, deltaACE2; see: https://www.uniprot.org/uniprot/Q9BYF1). With specific primers, we obtained signals for both the long and, to a lesser extent, the short/delta variant of ACE2 (data not shown). Our IF studies of ACE2 in term placenta were performed with an antibody raised against aa 19 to aa 740 of ACE2, and therefore not distinguishing between the short and the long splice variant. Our studies revealed a signal in the stromal compartment of the villi (Fig. 1). To further identify the cells, we performed double IF with various markers. As shown previously [Becker et al., 2020], collagen- and calcium-binding EGF domain 1 (CCBE1) is a marker of villous endothelial cells. We observed ACE2 in stromal cells, not in endothelial cells (Fig. 2). The cell junction molecule β-catenin, a key mediator of the WNT-signaling pathway, was expressed at the basal side of the syncytiotrophoblast, and again, ACE2 was found in stromal cells (Fig. 3). We then performed double-staining with antibodies against ACE2 and the macrophage marker CD68 and found double-positive cells (Fig. 4).

**Fig. 5:**
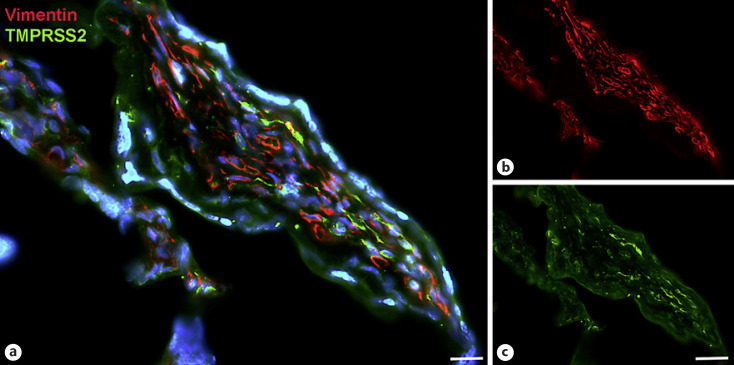
Immunofluorescence picture showing TMPRSS2 (green) and mesenchymal marker vimentin (red) in term placenta. **a** Merged picture; **b** and **c** show each channel separately. The two signals are found in stromal cells, but there is hardly any overlap. Nuclei are stained blue with DAPI. Bar = 20 µm in **a** and 40 µm in **b** and **c**.

**Fig. 6:**
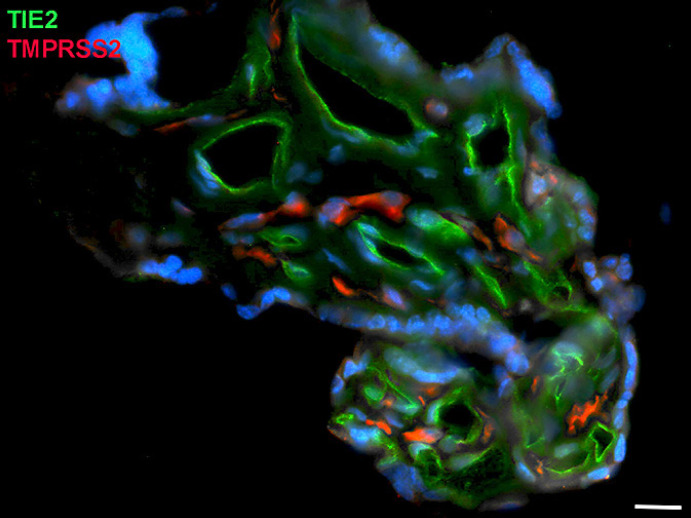
Immunofluorescence picture showing endothelial cell marker TIE2 (green) and TMPRSS2 (red) in term placenta. There is no overlap of the signals. Nuclei are stained blue with DAPI. Bar = 20 µm.

**Fig. 7:**
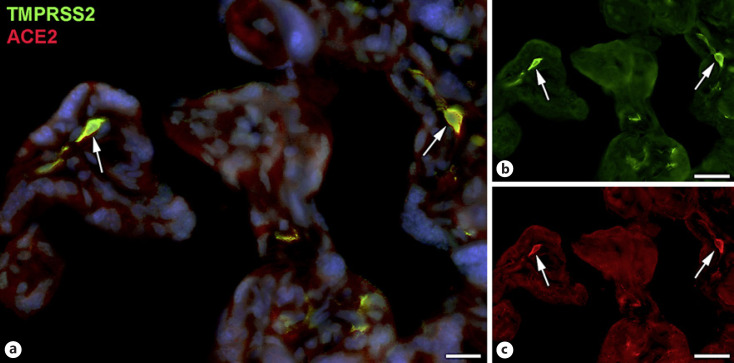
Immunofluorescence picture showing TMPRSS2 (green) and ACE2 (red) in term placenta. **a** Merged picture; **b** and **c** show each channel separately. A number of TMPRSS2+ stromal cells also express ACE2 (arrows). Nuclei are stained blue with DAPI. Bar = 20 µm in **a** and 40 µm in **b** and **c**.

**Fig. 8:**
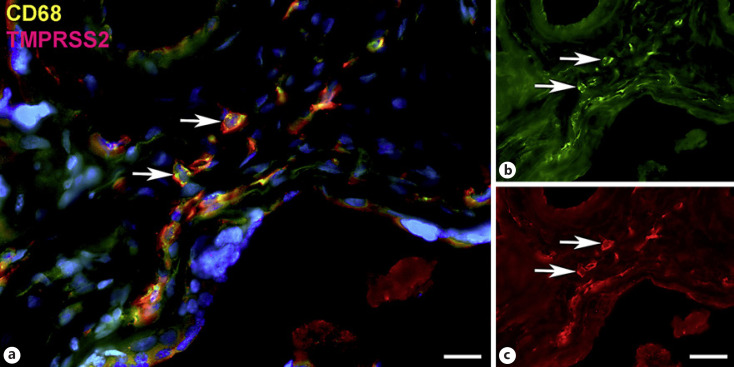
Immunofluorescence picture showing TMPRSS2 (red) and macrophage marker CD68 (green) in term placenta. **a** Merged picture; **b** and **c** show each channel separately. A subpopulation of TMPRSS2+ cells can be identified as macrophages (arrows). Nuclei are stained blue with DAPI. Bar = 20 µm in **a** and 40 µm in **b** and **c**.

**Fig. 9:**
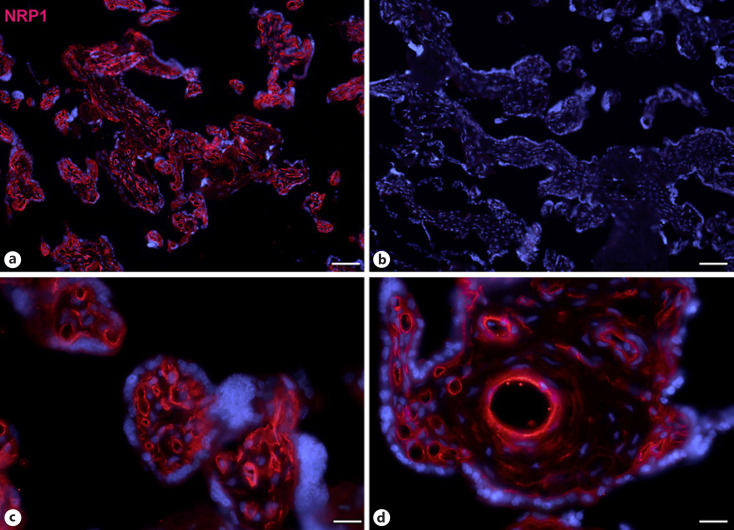
Immunofluorescence picture showing NRP1 (red) in term placenta. High NRP1 expression is found in endothelial cells. Nuclei are stained blue with DAPI. **b**Negative control without primary antibody. Bar = 80 µm in **a** and **b**, and 20 µm in **c** and **d**.

Next, staining with antibodies against TMPRSS2 also revealed positive cells in the stroma. Double-staining with the mesenchymal marker vimentin showed that there was almost no overlap of the two signals (Fig. 5). Double staining with antibodies against the endothelial marker TIE2 (TEK tyrosine kinase) confirmed TMPRSS2 expression in stromal cells, not in endothelial cells (Fig. 6). Double staining against TMPRSS2 and ACE2 revealed cells in the stroma, which expressed TMPRSS2 and ACE2 cells (Fig. 7). Furthermore, double-staining for TMPRSS2 and CD68 revealed co-expression in macrophages (Fig. 8). Staining for NRP1 revealed a strong signal on endothelial cells, but no signal at the apical surface of the syncytiotrophoblast (Fig. 9).

## Discussion

We studied the expression of COVID19 receptors in term placenta. We observed immunopositivity for ACE2 and TMPRSS2 in stromal cells. A subpopulation of the cells stained positive for CD68, and can therefore be designated as macrophages/Hofbauer cells. This is in line with the finding of SARS-CoV-2 RNA in CD14^+^ mononuclear cells in placental villi of COVID-19^+^ women [Facchetti et al., 2020]. By qPCR, we observed expression of the long and the short (delta) isoform of ACE2. It was shown recently that the truncated isoform can be induced by interferons and rhinoviruses, but not by SARS-CoV-2 infection [Onabajo et al., 2020; Blume et al., 2021]. These studies also showed that delta-ACE2 does not bind the COVID-19 spike protein and does not possess carboxypeptidase activity. As noted above, the antibody used does not distinguish between the truncated and the long form of ACE2. Nevertheless, expression in stromal cells, which are protected by the placental barrier, suggests that vertical transmission may be unlikely under physiological conditions. The specimens studied here were collected before the start of the COVID-19 pandemic from healthy women. We are therefore confident that the expression pattern described here is the physiological one. Its alteration during infection, however, cannot be ruled out.

We found expression of NRP1, a typical endothelial receptor [Shraga-Heled et al., 2007], in endothelial cells of placental vessels. None of the three investigated COVID19 receptors/coreceptors was found on the apical surface of the syncytiotrophoblast, which is the only physiological contact site for maternal blood in term placenta. Of note, we also studied ACE2 expression on paraffin sections with antigen retrieval methods (data not shown). This produced background staining, e.g. in nuclei in control sections, which appeared to be enhanced by the primary antibody.

With scRNASeq, expression of ACE2 was found in 9 out of 9,852 placenta-derived cells. These cells were also GATA3-positive and therefore classified as trophoblast cells; leaving open, which type of trophoblast cell they were exactly [Lü et al., 2020]. Single-cell RNA analyses of syncytiotrophoblast are, for obvious reasons, difficult to interpret. Also, the localization of the protein in the cell (apical vs. basal) must be determined. There are a number of studies which argue against vertical transmission during pregnancy, while others show ACE2 immunopositivity in syncytiotrophoblast (an overview of the literature can be found, e.g. in [Verma et al., 2021; Linehan et al., 2021]. Thereby, ACE2 expression was usually shown in placentas from COVID-19^+^ women, but coexpression of TMPRSS2 was not detected [Taglauer et al., 2020]. SARS-CoV-2 infection in syncytiotrophoblast was detected, e.g. with immunostaining against the viral nucleocapsid or spike protein [Facchetti et al., 2020; Hsu et al., 2021]. However, COVID-19 positivity in the syncytiotrophoblast does not necessarily correlate with viral infection of the fetus [Penfield et al., 2020]. In 2 out of 22 neonates described by Patane et al., COVID-19-positivity was observed in syncytiotrophoblast in addition to histiocytic intervillositis, associated with macrophages in both the intervillous and villous spaces [Patane et al., 2020].

## Conclusion

Recent data provide evidence that in a certain number of neonates (around 13%) born to COVID-19^+^ women vertical transmission does occur [Villar et al., 2021]. A very recent study confirms the high risks of SARS-CoV-2 infection for the pregnant women and their fetuses, but it also emphasizes that “there were no additional adverse neonatal outcomes, other than those related to preterm delivery” [Gurol-Urganci et al., 2021]. Our data on healthy term placenta seem to show that the placenta provides a barrier against vertical transmission of COVID-19. The main receptors for viral entry, TMPRSS2 and ACE2, are not detectable by IF on the apical surface of the syncytiotrophoblast. Stromal cells, which in part are CD68^+^ Hofbauer cells, seem to be most susceptible for COVID-19 infection by expressing TMPRSS2 and ACE2. Virus propagation via circulating mononuclear cells was proposed earlier [Facchetti et al., 2020]. Microtrauma during placental development (which is the most likely reason for fibrinoid deposits), as well as placentitis [Linehan et al., 2021], and probably cesarian resection [Villar et al., 2021] may also be routes for vertical transmission of SARS-CoV-2. However, further viral receptors and coreceptors, such as TMPRSS4 [Zang et al., 2020], must be taken into consideration.

## Statement of Ethics

Placentas were collected after vaginal births of healthy children with the informed, written consent of the mothers. The studies were approved by the ethics committee of the University Medical Hospital Göttingen, UMG (application No.: 18/1/18).

## Conflict of Interest Statement

The authors declare that they have no conflicts of interest related to the research.

## Funding Sources

We did not receive specific funding to conduct this study.

## Author Contributions

Jürgen Becker: data curation, supervision. Danny Qiu: data acquisition, writing − draft. Walter Baron: material acquisition and patient education. Jörg Wilting: conceptualization, data curation, supervision, writing - review and editing.

## Data Availability Statement

All immunofluorescence data are included in the article. Real-time PCR data will be presented upon request.

## Figures and Tables

**Table 1:** Antibodies used in this study

	Dilution	Manufacturer	Lot
*Primary antibody*			
β-Catenin, mouse-anti-human	1:500	Biosciences, Franklin Lakes, USA	610153
CCBE1, rabbit-anti-human	1:500	Sigma-Aldrich, Munich, Germany	R38605
CD68, mouse-anti-human	1:10	Dako, Jena, Germany	20055015
ACE-2, mouse-anti-human	1: 100	ReliaTech, Wolfenbüttel Germany	11A31
TMPRSS2, rabbit-anti-human	1:100	Abcam, Cambridge, UK	GR3343160-1
NRP1, rabbit-anti-human	1:100	ReliaTech, Wolfenbüttel Germany	1403R16
TIE2, mouse-anti-human	1:100	ReliaTech, Wolfenbüttel Germany	0512R09
Vimentin, mouse-anti-human	1:10	Dako, Jena, Germany	20035381
*Secondary antibody*			
IgG H + L, goat-anti-rabbit Alexa 488	1: 200	Invitrogen, Waltham, USA	A11008
IgG H + L, goat-anti-mouse Alexa 594	1: 200	Invitrogen, Waltham, USA	A11008
IgG1, goat-anti-mouse Alexa 488	1: 200	Invitrogen, Waltham, USA	A21121
IgG2a, goat-anti-mouse Alexa 594	1: 200	Invitrogen, Waltham, USA	2044860

**Table 2:** Primers used for qPCR

Genes	Primer name	Sequence
ACE2	ACE2_fwd ACE2_rev	AGAAAGCAGTCTGCCATCCC GCTGTCAGGAAGTCGTCCAT

TMPRSS2	TMPRSS2_fwd TMPRSS2_rev	ATACAAGCTGGGGTTCTGGC AGACCATGTGGATTAGCCGT

β-Actin	Actin_fwd Actin_rev	ATTGGCAATGAGCGGTTC TGAAGGTATTTCGTGGATGC

ACE2 total	totACE2_fwd totACE2_rev	TGGGACTCTGCCATT TACTTAC CCCAACTATCTCTCG CTTCATC

ACE2 long isoform	lonACE2_fwd lonACE2_rev	CAAGAGCAAACGGTTGAACAC CCAGAGCCTCTCATT GTAGTCT

ACE2 short isoform	shoACE2_fwd shoACE2_rev	GTGAGAGCCTTAGGT TGGATTC AAGGATCCTCCCTC CTTTGT
